# Infectious Disease Awareness Among Future Health Professionals: A Comparison of Knowledge, Attitudes, and Practices Between Nursing Students in Japan and Laos

**DOI:** 10.3390/pathogens14090920

**Published:** 2025-09-11

**Authors:** Hansani Madushika Abeywickrama, Tiengkham Pongvongsa, Marcello Otake Sato, Rie Takeuchi, Yoichi Abiko, Tippayarat Yoonuan, Jun Kobayashi, Megumi Sato

**Affiliations:** 1 Graduate School of Health Sciences, Faculty of Medicine, Niigata University, Niigata 951-8518, Japan; hansani@clg.niigata-u.ac.jp; 2 Savannakhet Provincial Health Department, Savannakhet 13000, Laos; tiengkhampvs@gmail.com; 3 Division of Global Environment Parasitology, Faculty of Medical Technology, Niigata University of Pharmacy and Medical and Life Sciences, Niigata 956-8603, Japan; 4 Faculty of Medicine, International University of Health and Welfare, Narita 286-0048, Japan; zhuneilihui@gmail.com; 5 Naka-Kinen Clinic, Nakadai, Naka 311-0113, Japan; okiba437@gmail.com; 6 Department of Helminthology, Faculty of Tropical Medicine, Mahidol University, Bangkok 10400, Thailand; tippayarat.yoo@mahidol.edu; 7 Department of Global Health, Graduate School of Health Sciences, University of the Ryukyus, Nishihara 903-0215, Japan; junkoba@med.u-ryukyu.ac.jp

**Keywords:** infectious diseases, neglected-tropical diseases, zoonoses, knowledge attitudes and practices, nursing students, Japan, Laos

## Abstract

This study assessed and compared the knowledge, attitudes, and practices of nursing students in Japan and Laos, two countries with distinct epidemiological and socioeconomic contexts, regarding neglected tropical diseases (NTDs) and zoonoses. A cross-sectional survey was conducted using a self-administered questionnaire among 190 nursing students from Japan and 254 students from Laos. Descriptive statistics, chi-square test, and Fisher’s exact test were used to analyze intergroup differences. Most of the study participants were female and in their first or second year of their nursing program. Knowledge gaps were identified in both groups. Japanese students showed a higher perceived susceptibility to infections and greater adherence to hygiene practices. Laotian students reported higher exposure to endemic diseases and more frequent contact with livestock. Attitudes toward public health education were generally positive, although Japanese students expressed a greater willingness to engage in future educational roles. However, gaps between attitude and actual practice were apparent in both groups, including inconsistent use of soap and insect repellents. The findings reveal a gap in knowledge of NTDs and zoonoses and a deficit in translating knowledge and attitudes into preventive behaviors. Findings highlight the need for tailored educational strategies considering behavioral and cultural factors to strengthen the nurse’s role in infectious disease prevention.

## 1. Introduction

Infectious diseases (IDs), caused by pathogenic microorganisms, such as bacteria, viruses, parasites, or fungi, which spread directly or indirectly from one person to another, continue to pose a significant health burden worldwide [1]. Emerging and re-emerging outbreaks have renewed the focus on IDs despite the decline in associated morbidity and mortality rates owing to improvements in sanitation conditions and healthcare access, and medical advances [2,3]. Additionally, IDs incur significant economic and social costs, underscoring the need to intensify prevention and control efforts in this area.

Neglected tropical diseases (NTDs) are a subset of approximately 20 IDs, including dengue, chikungunya, foodborne trematodiases, soil-transmitted helminthiases (STHs), and leishmaniasis [4,5]. According to the WHO, over 1 billion people worldwide are affected by NTDs, primarily in disadvantaged communities in tropical and subtropical regions, mainly because of limited access to clean water, poor sanitation, and inadequate healthcare infrastructure [5]. Zoonoses are infectious diseases transmitted from animals to humans, often through close contact with pets or livestock [6]. Together, Zoonoses and NTDs constitute a significant portion of both newly discovered and existing IDs [2]. Public health control of NTDs and zoonoses poses a challenge due to their complex epidemiology, vector-based transmission, animal reservoirs, and intricate environmental interactions and life cycles [4]. Effective management of IDs requires a knowledgeable health workforce, and nurses, in particular, play a key role in NTD prevention and health education [7].

The knowledge, attitude, and practice (KAP) model suggests that an individual’s knowledge about a particular subject can shape their attitudes, which subsequently influence their behaviors and decisions. Furthermore, people are more likely to engage in beneficial actions if they have accurate information and favorable attitudes [8]. Thus, nursing students, as future healthcare providers, must be equipped with sound foundational KAP to manage IDs and mitigate transmission.

This study assessed and compared the KAP regarding selected IDs among nursing students in Japan and Laos, two countries with markedly different socioeconomic and developmental profiles. Japan, a developed country with a Human Development Index (HDI) of 0.925 (2022), contrasts sharply with Laos, a lower-middle-income country with an HDI of 0.617 [9]. These macro-level differences are mirrored in healthcare infrastructure and education. Local disease epidemiology, patient profiles, and at-risk populations also differ substantially between the two countries. For example, indigenous malaria transmission has been eliminated in Japan since 1961 [10], whereas malaria remains a continuing public health concern in Laos [11]. These contextual differences likely shape students’ experiences, clinical exposures, and learning priorities, contributing to the differences in KAP between the two countries.

By comparing nursing students’ KAP in two countries with such contrasting characteristics, this study aimed to explore how developmental disparities influence the understanding and behavior of individuals within the same professional training domain. Assessing baseline KAP among nursing students, rather than experienced nurses, improves cross-country comparability by reducing heterogeneity in clinical experience, specialty, and institutional policies, and allows identification of educational and training gaps at the formative stage before workplace exposure. In doing so, the study sought to identify gaps, inform future education strategies, and contribute to more context-sensitive public health interventions.

## 2. Materials and Methods

### 2.1. Study Design and Setting

A cross-sectional survey was conducted in February 2018 at selected nursing schools in Japan (Niigata and Okinawa) and Laos (Savannakhet Province).

### 2.2. Study Sample and Procedures

The sample size was calculated using Cochran’s formula with a 5% margin of error, 95% confidence interval, and 50% expected awareness of NTDs (due to the absence of prevalence data), resulting in a minimum of 384 respondents. After accounting for potential attrition and non-response, the sample size was increased by 4%, and a total of 400 nursing students from each country were invited through the distribution of an information sheet and questionnaire. Returning the completed questionnaire was considered as implied consent.

Ethical approval for the study was granted by the Ethical Review Committee on Research involving Human Subjects at Niigata University, Japan (No. 2017-0140) and the National Ethics Committee for Health Research, National Institute of Public Health, Ministry of Health, Laos (No. 037/NIOPH/NECHR). The study was conducted in accordance with the Declaration of Helsinki of the World Medical Association and reported according to the Strengthening the Reporting of Observational Studies in Epidemiology (STROBE) guidelines for cross-sectional studies (Supplementary Materials S1) [12].

### 2.3. Study Instrument

#### 2.3.1. Questionnaire Development and Validation

A self-administered questionnaire was developed specifically for this study, following a multistage process, due to the lack of a validated and standardized questionnaire for assessing KAP on multiple NTDs or IDs at the time. While some items in the questionnaire were adapted from existing sources [13,14,15], others were newly created based on WHO guidelines and country-specific disease priorities.

An expert panel of public health specialists and nursing educators from both countries reviewed the initial draft for content validity and cultural appropriateness. The questionnaire, initially developed in English, was translated into Japanese and Lao (the national languages of the two countries) using a forward–backward translation process by bilingual experts familiar with public health terminology. A pilot test was conducted with 20 nursing students (~5% of the sample) from each country to assess clarity and comprehension, informing minor revisions in wording and structure.

#### 2.3.2. Questionnaire Content

The final questionnaire comprised 50 items across 4 domains (Supplementary Materials S2). The demographic variables included sex, age, and academic year. The knowledge domain assessed the awareness of symptoms and transmission of selected IDs, including strongyloidiasis, angiostrongyliasis, toxoplasmosis, leptospirosis, Japanese encephalitis (JE), dengue, malaria, STHs, and tick-borne diseases (TBDs). In addition, the diseases were adapted per the public health relevance in each country and the pathogen’s habitat; for example, taeniasis (beef tapeworm) in Laos and diphyllobothriasis (fish tapeworm) in Japan; opisthorchiasis (liver fluke) in Laos and anisakis in Japan. The section also covered methods of learning basic hygiene techniques (BHTs) and the sources of information on IDs.

The attitude domain (19 items) assessed perceptions of disease prevention, modes of transmission, and the value of health education. Students were also asked which diseases should receive prioritized attention in their residential area and in the future. The practice domain (21 items) inquired about self-reported preventive behaviors such as washing uncooked vegetables; hand hygiene (before eating, after using the toilet, and after touching pets or their feces); limiting skin exposure in vector-prone areas; using insect repellents; and avoiding walking barefoot outside.

### 2.4. Statistical Analysis

Descriptive statistics are presented as frequencies (*n*) and percentages (%). As the response rates varied across the items, proportions were calculated using the number of valid responses per item. Differences between the two countries were assessed using chi-square or Fisher’s exact test, with significance set at *p*-value < 0.05. Analyses were performed using SPSS 27.0 statistical software (IBM Inc., Tokyo, Japan), and the figures were generated using RStudio (version 2025.05.1+513.pro3).

## 3. Results

A total of 190 Japanese (response rate: 47.5%) and 254 Laotian (response rate: 63.5%) nursing students participated. No individual-level data were collected from non-respondents, as non-response was considered non-consent; therefore, analysis of non-response bias was not possible. In Japan, 84.7% of the students were female, with the academic year distribution as follows: fourth year (33%), second year (34%), and first year (30%). Laotian students included 52 nursing and 23 midwifery students (all in the second year), and 179 primary healthcare students (39.1% in the first year, 25.1% in the second year, and 35.8% in the third year.

### 3.1. Knowledge of IDs

Table 1 compares disease-specific awareness between nursing students in Japan and Laos. Laotian students were more aware of parasitic and helminthic infections such as strongyloidiasis, opisthorchiasis, taeniasis, and STHs, while Japanese students showed higher awareness of vector-borne diseases like dengue, malaria, and JE. Among those aware of the disease, selecting angiostrongyliasis, toxoplasmosis, taeniasis/diphyllobothriasis, leptospirosis, JE, TBDs, and opisthorchiasis/anisakis was counted as correct identification of a zoonotic disease. As shown in Table 1, percentages correctly identifying zoonoses were generally low in both countries. By contrast, a substantial percentage of students misclassified dengue and malaria as zoonotic despite high awareness.

**Figure 1:**
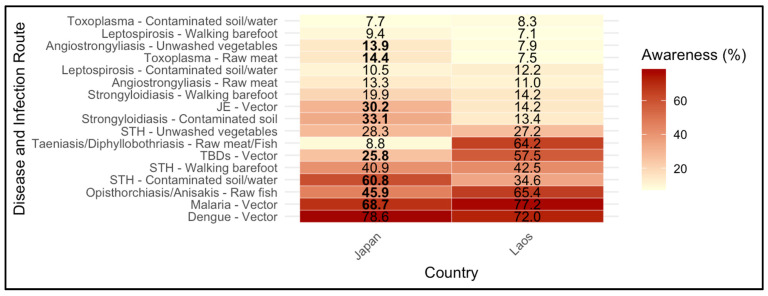
Awareness of infectious disease transmission routes among nursing students in Japan and Laos (*n* = 181). The heatmap shows the percentage of students who correctly identified the main infection route/s for each disease. Columns are two countries (Japan, Laos), rows list disease-route pairs, cell values represent percentages (%), and the color bar is scaled 0–80% (darker colors represent higher awareness). Significant differences between countries were tested using the chi-square test or Fisher’s exact test, as appropriate, and statistical significance is indicated in bold (*p* < 0.05). Awareness of infection route was higher for angiostrongyliasis-unwashed vegetables, toxoplasma-raw meat, JE-vector, strongyloidiasis-contaminated soil, and STH-contaminated soil/water among Japanese students, while it was higher for TBDs-vector and malaria-vector among Laotian students. Taeniasis (raw meat) and Opisthorchiasis were included only in the Laos questionnaire. Diphyllobothriasis (raw fish) and Anisakis were included only in the Japanese questionnaire. Abbreviations: JE = Japanese encephalitis, TBDs = Tick-borne diseases, STH = Soil-transmitted helminthiases.

Awareness of infection route and symptoms was significantly different between Japanese and Laotian students. Laotian students demonstrated a greater awareness of transmission routes for endemic parasitic infections, such as taeniasis (64.2% vs. 8.8%) and opisthorchiasis (65.4% vs. 45.9%), while both groups frequently identified vectors as the transmission route for dengue and malaria. Awareness was significantly higher among Japanese students for angiostrongyliasis-unwashed vegetables (13.9 vs. 7.9%), toxoplasma-raw meat (14.4% vs. 7.5%), JE-vector (30.2% vs. 14.2%), strongyloidiasis-contaminated soil (33.1% vs. 13.4%), and STH-contaminated soil/water (60.8% vs. 34.6%) disease-route pairs (Figure 1).

Symptom recognition was higher among Japanese students for JE, dengue, and malaria. Overall, knowledge of transmission and symptoms was low among both groups, except for well-known diseases such as dengue and malaria (Table 2).

**Figure 2:**
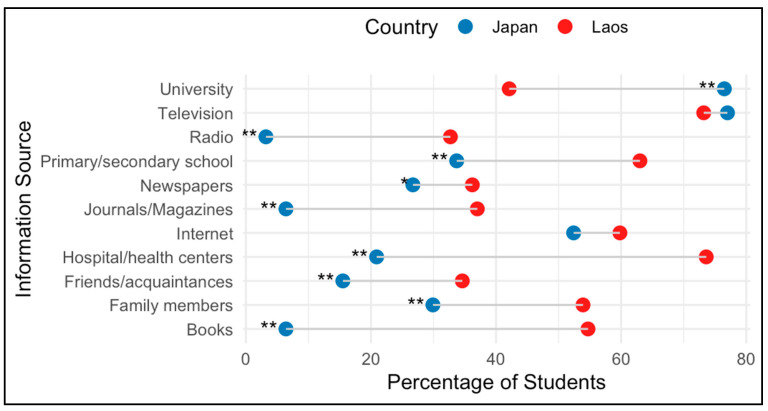
Sources of information about infectious diseases among nursing students in Japan (*n* = 187) and Laos (*n* = 254). Blue (Japan) and red (Laos) points indicate the percentage of students reporting each source (multiple answers allowed). Statistically significant differences between groups were assessed using the chi-square test or Fisher’s exact test (* *p* < 0.05, ** *p* < 0.001).

Regarding knowledge of BHTs, such as hand washing and gargling, most Japanese students reported learning from family (72/132), followed by kindergarten (*n* = 33) and primary school (*n* = 19). In comparison, 133 and 82 out of 254 Laotian students reported learning from family and primary school, respectively. Japanese students cited television (76.9%), university lectures (76.5%), and the Internet (52.8%) as their primary sources of information on IDs, while over 70% of Laotian students mentioned hospitals, health centers, and television. Radio, journals/magazines, and books were reported by less than 10% of Japanese students, which was significantly lower compared to Laotian students (Figure 2).

### 3.2. Attitudes

Table 3 shows significant differences in attitudes among Japanese and Laotian students. Most Japanese students perceived themselves as susceptible to IDs and were more concerned about transmission. Both groups reported high levels of precautionary behaviors in clinical settings, with slightly higher agreement among Japanese students. Most students valued the knowledge of IDs and health education; however, attitudes toward the impact of health education on public perceptions varied, with Japanese students showing greater agreement than their Laotian counterparts (87.2% vs. 44.9%). Despite this, the willingness to engage in public health education was low among Japanese students (75.5%) compared to Laotian students (85.8%).

**Figure 3:**
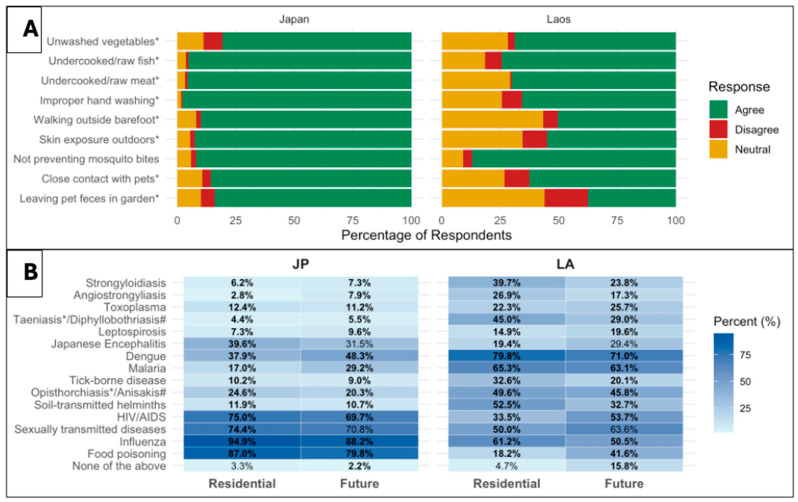
Attitudes toward transmission routes and perceived importance of infectious diseases among nursing students in Japan (JP) and Laos (LA). **Panel** (**A**): Attitudes toward infectious disease transmission routes among nursing students in Japan and Laos. Responses are shown as stacked proportions of ‘Disagree,’ ‘Neutral,’ and ‘Agree,’ calculated based on the total number of responses for each item (Japan: 187–188; Laos: *n* = 254). Statistically significant differences between countries were identified using the chi-square test or Fisher’s exact test (*p* < 0.05) and are indicated with an asterisk (*) next to the raw label. **Panel** (**B**): Perceived importance of infectious diseases in the residential area and in the future. The heatmap shows the percentage of students selecting each disease as a disease that should be given priority in their residential area and in the future (multiple responses were allowed). Darker shades represent higher proportions of students selecting the disease. Values in bold indicate statistically significant differences between the two countries, identified using the chi-square test or Fisher’s exact test (*p* < 0.05). * Item included only in the Laos questionnaire, # Item included only in the Japanese questionnaire.

Perceptions of infectious disease transmission routes varied notably between Japanese and Laotian nursing students (Figure 3, Panel A). While most students correctly identified common transmission routes (improper hand washing, undercooked meat or fish, and unwashed vegetables), Japanese students showed consistently higher. For instance, over 95% of Japanese students believed infections could result from undercooked meat or fish, compared to 70.1% and 74.4% of Laotian students, respectively. Laotian students showed greater uncertainty or disagreement on skin exposure, barefoot walking, and pet-related risks. The only response with no significant difference between the two groups was mosquito-borne transmission (*p* = 0.246).

Regarding priority diseases in their residential areas and in the future, Japanese students selected influenza, food poisoning, and sexually transmitted infections (STIs), while Laotian students more frequently chose vector-borne and parasitic diseases. Most differences were statistically significant, reflecting distinct disease burdens (Figure 3, Panel B).

### 3.3. Preventive Practices

#### 3.3.1. Consumption of Uncooked Vegetables

Both groups consumed uncooked vegetables 2–3 times per week (Japan: 63.1% [*n* = 187], Laos: 59.8% [*n* = 254]), while daily consumption was higher among Laotian students (20.5% vs. 10.7%). A small proportion of students reported not eating uncooked vegetables at all (Japan: 6.4%, Laos: 11.4%). Most students washed vegetables with tap water (Japan: 87.6% [*n* = 170]; Laos: 96.4%), although pooled water use was more common among Japanese students (11.2% vs. 1.3%). A few reported not washing vegetables before consumption (Japan: 0.6%, Laos: 2.2%).

Lettuce (*Lactuca sativa*) was the most consumed uncooked vegetable in Japan (>90%), followed by spring onion (*Allium* spp., 43.0%). In contrast, Laotian students consumed a wider variety of vegetables uncooked, including spring onion (63.6%), followed by hoary basil (*Ocimum americanum*, 42.7%), coriander (*Coriandrum sativum* L., 41.8%), and holy basil (*Ocimum tenuiflorum*, 40.4%).

#### 3.3.2. Raw Meat and Fish Consumption

The consumption of raw or undercooked meat was higher among Laotian students than among Japanese students (153/254 vs. 65/189), with 52 reporting a frequency of 2–3 times per week and 64 reporting a frequency of 2–3 times per month. Beef was the most commonly consumed raw or undercooked meat in both groups (Japan: 21/65 vs. Laos: 54/153).

In contrast, Japanese students more often ate raw fish (168/187 vs. 114/254), with 68% consuming it 2–3 times per month. Among fish eaten raw, Japanese students most frequently consumed ‘Mackerel’ (*n* = 49), while many Laotian students consumed *Esomus metallicus* (*n* = 109).

#### 3.3.3. Vector Exposure and Animal Contact

The use of insect repellent was higher among Japanese students (74.7% vs. 52.2%, *p* < 0.001). By activity, Japanese students reported using repellent during hiking (*n* = 105), camping (*n* = 101), farming (*n* = 36), and gardening (*n* = 59), while the respective proportions for Laotian students were (*n* = 65, 50, 33, and 45, respectively). Avoidance of mosquito bites was the primary reason for repellent use (Japan: *n* = 162; Laos: *n* = 226). Tick bites were reported by 45 Japanese and 66 Laotian students, while 121 Laotian students reported daily mosquito bites.

A significantly higher percentage of Laotian students reported walking barefoot outdoors (69.3% vs. 23.5%) and limiting skin exposure outside (89.0% vs. 76.6%) compared to Japanese students (both *p* < 0.001). Pet ownership was higher among Laotian students (134/251) than Japanese students (66/184), with dogs being the most common pet among both groups (Japan: *n* = 46; Laos: *n* = 105). Additionally, 53 Japanese and 96 Laotian students disposed of pet feces as regular garbage. Livestock ownership was reported only by Laotian students (*n* = 83), with cows (*n* = 59) and chickens (*n* = 58) being the most common animals. Notably, 67 Laotian students reported using manure as fertilizer.

Table 4 summarizes the hand washing practices in the daily lives of Japanese and Laotian nursing students. Japanese students were more likely to use soap before eating (55.6% vs. 34.3%, *p* < 0.001) and after cleaning pet feces (80.9% vs. 61.4%, *p* = 0.014), except after touching pets, where Laotian students more often used soap.

## 4. Discussion

The present study explored and compared the KAP regarding IDs among nursing students from Japan and Laos, two countries with contrasting socioeconomic development, health infrastructure, disease epidemiology, and disease burden. This study offers insights into how diverse national contexts impact KAP patterns among future healthcare professionals. Rather than seeking direct equivalence, this study acknowledges these differences to illuminate gaps and opportunities for strengthening infection control education globally.

This study focused particularly on NTDs and zoonoses, which are often understudied, while considering the differing disease epidemiologies in these two countries. Previous KAP studies have typically been limited to a single region or a disease, with few cross-country comparisons across multiple IDs. Thus, our findings are both unique and valuable, revealing how nursing students’ perceptions and behaviors are shaped by local epidemiology, educational exposure, and cultural norms.

### 4.1. Knowledge of IDs

The majority of participants from both countries had heard of dengue and malaria (Table 1). While both are predominantly imported IDs in Japan [16], they are endemic and major public health concerns in Laos [17]. A previous study reported low malaria awareness among Japanese travelers, characterized by poor preventive practices, limited knowledge of symptoms, and unawareness of destination-specific risks [18]. In contrast, the high awareness of Japanese students about dengue and malaria in the present study may be attributed to the successful educational and health promotion campaigns. Many Laotian students recognized malaria and dengue as diseases that require particular attention in their residential areas (Figure 3); thus, the endemic nature of these diseases in their communities likely explains their greater awareness.

The patterns of knowledge and awareness varied between countries in ways consistent with local epidemiology and occupational exposures. In Laos, higher awareness of opisthorchiasis and STHs reflects their ongoing public-health relevance. Transmission of *Opisthorchis viverrini* is linked to the frequent consumption of raw or undercooked freshwater fish in Laos [19,20]. In Japan, an estimated 20,000 cases of anisakiasis occur annually [21], yet awareness among Japanese students was low, despite substantially higher consumption of raw/undercooked fish (89.8%), indicating a gap between exposure-relevant behaviors and disease knowledge. In Laos, STHs remain prevalent [22], notably *Ascaris lumbricoides*, *Trichuris trichiura*, and hookworms (*Ancylostoma duodenale*, *Necator americanus*, *Ancylostoma ceylanicum*) [23], which aligns with greater recognition of STH-related risks. By contrast, in Japan, STHs such as ascariasis, ancylostomiasis/necatoriasis, and trichuriasis are now rarely reported, likely resulting in a reduction in public health emphasis and clinical exposure. These contextual differences plausibly shape students’ experiences, clinical encounters, and learning priorities, contributing to cross-country differences in knowledge.

A notable share of students who classify malaria and dengue as zoonotic diseases points to a gap in foundational concepts, where many students appear to confuse zoonosis with vector-borne transmission. Overall, the lack of awareness of zoonoses in both groups observed in this study is concerning, given their global significance. The predominance of first- and second-year students in our sample may partly explain the knowledge gaps. A recent study reported higher knowledge of NTDs and control strategies among medical students than non-medical students in five Asian countries [24], while other studies reported knowledge gaps among medical and nursing students [25,26].

### 4.2. Attitudes Toward Infection Prevention and Health Education

There was a significant difference in attitudes toward IDs between nursing students from the two countries. Japanese students exhibited greater perceived susceptibility to IDs, with over 80% expressing concern about all the transmission routes inquired in the survey, reflecting a heightened risk perception. Risk perception, awareness, or belief regarding the likelihood and severity of potential harm significantly influences health-related behaviors; thus, it is vital in the context of ID control [27,28]. This likely explains the higher rates of hygiene and infection control behaviors among Japanese students (Table 4), consistent with prior studies that linked risk perception to prevention [28,29,30]. However, excessive risk perception or fear of IDs may negatively impact nursing students’ professional development [31,32], highlighting the need for education programs to strike a balance between providing updated knowledge of IDs and minimizing fear-driven attitudes [32].

In contrast, Laotian students had a lower risk perception but still reported strong preventive practices, suggesting that factors beyond risk perception, such as education, institutional policies, or cultural norms, may influence effective preventive behaviors. Previous studies have emphasized the importance of structured infection control education in promoting adherence to precautionary behavior [33,34], highlighting the need for educational programs that focus on underrepresented topics, such as NTDs.

A majority of Japanese students exhibited favorable attitudes toward health education on IDs, and willingness to participate in public health education once they become nurses. This is a positive sign, given the vital role of nurses in disseminating health information and promoting preventive behaviors within communities [35,36]. Laotian students were less optimistic about the impact of health education, potentially due to differing perceptions of the nurse’s role in society, public health infrastructure, trust in healthcare systems, and limited exposure to community-based education during training [37]. Prior studies have also reported mixed willingness among healthcare students to engage in NTDs control activities [24,25,26], highlighting the need for culturally relevant curricula in nursing education.

### 4.3. Preventive Practices in Daily Life and Clinical Settings

Both groups reported good hand-washing practices, although soap use was inconsistent, and some Japanese students neglected handwashing after pet contact. Inadequate hand hygiene, particularly the omission of soap, is a well-established contributor to the transmission of IDs [38,39]. However, it is worth noting that this study was conducted before the COVID-19 pandemic, during which public awareness and compliance with hand washing were globally emphasized [40,41].

Although 50.4% of Laotian students believed that walking barefoot increased the risk of infection, over 60% of the students were non-compliant. Similarly, 47% did not use insect repellent in high-risk settings. This discrepancy between beliefs and practices may stem from a limited understanding of transmission routes or low perceived personal risk observed among Laotian students. These findings underscore the need for targeted interventions to bridge the knowledge-practice gap.

Both groups reported learning BHTs at an early stage, primarily from family, followed by nursery or primary school. Family members were recognized as the most effective source of learning BHT. Existing research emphasizes the crucial role of family in shaping children’s hygiene habits, which are often acquired through observation and repetition [42]. Early-life interventions are recommended to foster lasting behavioral changes [43]. The high rates of hand-washing practices reported in this study suggest that early BHT education has been translated into sustained hygiene practices.

Television and the Internet were the two primary sources of ID-related information for students from both countries, highlighting the importance of digital media in health education, particularly in reaching younger populations. Prior studies found social media to be a key source for NTD knowledge among healthcare students [25,26]. These trends emphasize the importance of providing accurate and accessible content on digital platforms to enhance public health communication [44].

### 4.4. Implications

The findings of this study have implications for nursing education and for the roles nurses play in patient care and public health in both countries. In Japan, hospital-based nurses typically work within multidisciplinary teams with ready access to specialist consultation, advanced laboratory testing, and up-to-date epidemiological information, and public health nurses have roles in health education and surveillance, often working through municipal programs. In Laos, nurses in clinical settings provide first-line care under limited resources, including laboratory diagnostics and access to updated data, relying more on clinical judgment and experience when formal diagnostics are scarce. These contrasts likely contribute to the KAP gaps we observed in this study and imply different opportunities for nursing education, scope of practice, and population-level impact.

Therefore, nursing education and training programs should be aligned with local public health priorities, ensure access to up-to-date evidence, and utilize available resources optimally, such as laboratory tests and validated diagnostic criteria. In Laos, education and training programs should emphasize differential diagnosis, working with limited diagnostics, point-of-care testing, referral pathways, and surveillance or reporting protocols. In Japan, training programs can strengthen interprofessional coordination, surveillance and referral protocols, and community engagement skills that link bedside care and public-health programs.

Regarding research implications, Future studies would benefit from a standardized tool that captures a comprehensive picture of KAP, applicable across diverse cultural settings, and considers objective behavioral assessments. Furthermore, multinational studies involving diverse population groups (such as healthcare workers, agricultural workers, and fishery workers) would help clarify how national systems and local contexts influence KAP. Overall, these implications align with the WHO NTD Roadmap (2021–2030) [45] and related frameworks, such as ‘One Health’ [46] and WASH (Water, Sanitation, and Hygiene) [47], that emphasize integrated, multisectoral approaches to NTD prevention and control.

### 4.5. Limitations

This study has some limitations. First, no standardized tool has been validated to assess KAP across multiple IDs. Although our questionnaire was adapted from existing tools for specific IDs, such as dengue, and underwent review by an expert panel, translation, and pilot testing, formal psychometric validation procedures (factor analysis, reliability testing, test–retest analysis) were not conducted. Such validation requires a substantially larger sample size and a dedicated validation study, which was beyond the scope of this project. As our focus was on ensuring content validity, cultural appropriateness, and clarity of items, this approach was considered appropriate given the absence of a validated tool and the exploratory nature of this study. Second, the KAP tool used in this study does not capture comprehensive clinical competencies (diagnosis, principles, and methods of treatments, etc.) due to length and time constraints, and some questions were culturally specific, limiting direct comparison. Third, the sample was limited to selected institutions and may not be nationally representative. Fourth, the cross-sectional design does not capture temporal changes in KAP. Finally, reliance on self-reported data may have introduced response bias. We were unable to evaluate non-response bias because non-response was treated as non-consent, and no characteristics of non-respondents were available. Respondents may have been more interested in infectious diseases than non-respondents, potentially inflating KAP estimates; therefore, results should be interpreted with caution.

## 5. Conclusions

In conclusion, this study identified substantial knowledge gaps among nursing students in both countries, particularly regarding transmission and symptoms of IDs. Japanese students demonstrated higher risk perception, while both groups had positive attitudes toward public health education. However, practice gaps were observed, such as hand washing without using soap (both countries), neglecting hand washing after pet contact (Japan), walking barefoot outside, and not using repellents in risk environments (Laos). The findings suggest a disconnect between the students’ attitudes and their actual practices, highlighting the need for interventions to improve the knowledge and attitudes regarding less emphasized IDs such as NTDs, ensuring the translation of knowledge and attitudes into consistent practice. Medical and nursing education can benefit from the early integration of IDs into curricula, with emphasis on infection-control practices. Public health education programs can leverage digital media, particularly to reach the younger generation. Furthermore, education and prevention strategies should be culturally tailored and aligned with local epidemiological patterns.

## Figures and Tables

**Table 1:** Awareness of infectious and zoonotic diseases among Japanese and Laotian nursing students.

Infectious Disease	Which Infectious Diseases Have You Heard of?		What Are the Zoonotic Diseases?	
	Japan *n* = 190	Laos *n* = 254	Japan *n* = 166	Laos *n* = 254
Strongyloidiasis	15 (7.9)	115 (45.3) *	6 (3.6)	47 (18.5)
Angiostrongyliasis	9 (4.7)	43 (16.9) *	2 (1.2)	28 (11.0)
Toxoplasma	87 (45.8)	29 (11.4) *	13 (7.8)	11 (4.33) *
Taeniasis ^1^/Diphyllobothriasis ^2^	20 (10.5)	146 (57.5)	5 (3.0)	73 (28.7)
Leptospirosis	42 (22.1)	30 (11.8) *	14 (8.4)	5 (1.97)
JE	171 (90.0)	54 (21.3) *	44 (26.5)	9 (3.5) *
Dengue	174 (91.6)	195 (76.8) *	71 (42.8)	62 (24.4) *
Malaria	172 (90.5)	202 (79.5) *	64 (38.6)	91 (35.8)
TBDs	70 (36.8)	97 (38.2)	18 (10.8)	67 (26.4) *
Opisthorchiasis ^1^/Anisakis ^2^	83 (43.7)	170 (66.9)	30 (18.0)	79 (31.1)
STH	52 (27.4)	172 (67.7) *	22 (13.3)	87 (34.3)

Data are presented as numbers and proportions (%). Total percentages exceed 100% as multiple responses were allowed. The number of students who correctly identified the disease as zoonotic was calculated among respondents who reported having heard of that disease. * Significant differences between groups were identified using the chi-square test or Fisher’s exact test. ^1^ Only in the Laos questionnaire. ^2^ Only in the Japanese questionnaire. Abbreviations: JE = Japanese encephalitis, TBDs = Tick-borne diseases, STH = Soil-transmitted helminthiases.

**Table 2:** Knowledge of symptoms of infectious diseases among nursing students in Japan and Laos.

Symptoms	Japan *n* = 179	Laos *n* = 254	*p* Value *
Strongyloidiasis			
* Digestive symptoms*	58 (32.4)	79 (31.1)	0.775
* Skin symptoms*	31 (17.3)	28 (11.0)	0.060
Angiostrongyliasis			
* Fever*	34 (19.0)	4 (1.6)	<0.001
* Headache*	13 (7.3)	18 (7.1)	0.944
* Muscle pain*	10 (5.6)	12 (4.7)	0.687
Toxoplasma			
* Fever*	45 (25.1)	24 (9.4)	<0.001
* Muscle pain*	14 (7.8)	9 (3.5)	0.051
Taeniasis ^1^/Diphyllobothriasis ^2^			
* Digestive symptoms*	25 (13.8)	83 (32.7)	
Leptospirosis			
* Urinary symptoms*	22 (12.3)	9 (3.5)	0.001
JE			
* Fever*	162 (90.5)	173 (68.1)	<0.001
* Digestive symptoms*	58 (32.4)	118 (46.5)	0.003
* Headache*	35 (19.6)	56 (22.0)	0.530
* Muscle pain*	44 (24.6)	37 (14.6)	0.009
Malaria			
* Fever*	119 (66.5)	150 (59.1)	0.117
* Anaemia*	23 (12.8)	40 (15.7)	0.400
* Urinary symptoms*	14 (7.8)	9 (3.5)	0.051
TBDs			
* Fever*	38 (21.2)	64 (25.2)	0.338
* Skin symptoms*	36 (20.2)	67 (26.4)	0.140
Opisthorchiasis ^1^/Anisakis ^2^			
* Digestive symptoms*	72 (39.8)	33 (13.0)	
STH			
* Respiratory symptoms*	5 (2.8)	17 (6.7)	0.069
* Digestive symptoms*	47 (26.3)	82 (32.3)	0.177
* Anaemia*	11 (6.1)	11 (4.3)	0.397
* Skin symptoms*	53 (29.6)	45 (17.7)	0.004

Data are presented as numbers and proportions (%). * Significant differences between groups were identified using the chi-square test or Fisher’s exact test. ^1^ Only in the Laos questionnaire. ^2^ Only in the Japanese questionnaire. Abbreviations: JE = Japanese encephalitis, TBDs = Tick-borne diseases, STH = Soil-transmitted helminthiases.

**Table 3:** Proportion of students reported positive attitudes toward infectious disease prevention and health education among nursing students from Japan and Laos.

Attitude Statement	Japan, *n* (%) *	Laos, *n* (%) *	*p* Value #
1. Susceptible to infectious diseases in daily life	160 (86.0)	130 (51.2)	<0.001
2. Worried about contracting an infectious disease	174 (93.5)	198 (77.9)	<0.001
3. Take precautions to avoid contracting or spreading infectious diseases in daily life	135 (72.6)	176 (69.3)	0.003
4. Take precautions to avoid contracting or spreading infectious diseases during clinical training	175 (94.1)	200 (78.7)	<0.001
5. Having knowledge of infectious diseases is important	184 (98.4)	229 (90.1)	0.001
6. Health education on infectious diseases is important	182 (96.8)	224 (88.2)	0.001
7. Willing to educate the public on infectious disease prevention, once become a nurse	142 (75.5)	218 (85.8)	0.012
8. Health education change people’s attitudes toward infectious diseases	164 (87.2)	114 (44.9)	<0.001

Data are presented as numbers and proportions (%). * Proportions were calculated on the basis of the total number of responses for each item (Japan: response rates varied from 186 to 188, Laos: *n* = 254). # Significant differences between groups were identified using the chi-square test or Fisher’s exact test.

**Table 4:** Practice of hand washing in daily life by Japanese and Laotian nursing students.

	Japan *n* (%) *	Laos *n* (%) *	*p* Value ^#^
Hand wash before eating			
* Yes, only with water*	64 (34.2)	167 (65.7)	<0.001
* Yes, with soap*	104 (55.6)	87 (34.3)	
Hand wash after using the toilet			
* Yes, only with water*	86 (46.5)	130 (52.2)	0.382
* Yes, with soap*	97 (52.4)	114 (45.8)	
Hand wash after touching pets			
* Yes, only with water*	11 (16.2)	55 (41.7)	<0.001
* Yes, with soap*	28 (41.2)	72 (54.5)	
Hand wash after cleaning pets’ feces			
* Yes, only with water*	9 (13.2)	43 (33.9)	0.014
* Yes, with soap*	55 (80.9)	78 (61.4)	

* The proportions were calculated based on the total number of responses for each question. ^#^ Significant differences between groups were identified using the chi-square test or Fisher’s exact test.

## Data Availability

The data that support the findings of this study are available from the corresponding authors (MOS and MS), upon reasonable request.

## Supplementary Materials

The following supporting information can be downloaded at: https://www.mdpi.com/article/10.3390/pathogens14090920/s1, Supplementary Materials S1: STROBE guidelines for cross-sectional studies; Supplementary Materials S2: Questionnaire.

## Abbreviations

The following abbreviations are used in this manuscript:

BHT	Basic hygiene techniques
HDI	Human Development Index
IDs	Infectious diseases
JE	Japanese encephalitis
KAP	Knowledge, attitude, and practice
NTDs	Neglected tropical diseases
STIs	Sexually transmitted infections
STHs	Soil-transmitted helminthiases
TBD	Tick-borne diseases
