# Supplementary material for: Infectious Disease Awareness Among Future Health Professionals: A Comparison of Knowledge, Attitudes, and Practices Between Nursing Students in Japan and Laos
Source: Pathogens. 2025 Sep 11;14(9):920. doi: 10.3390/pathogens14090920 (PMC12472601; doi:10.3390/pathogens14090920)
Supplement: Supplementary file 1 [file pathogens-14-00920-s001.zip › S2 Supplementary document 1-Questionnaire.pdf]

Questionnaire - Laos

Date: day/   /month   /year

Please answer the following questions by checking (✓) the most appropriate response.
For most questions, select **only one answer**.
If a question is labeled **"Multiple answers allowed"**, you may choose **more than one option**.

Part I: Demographic information

1. Gender
   ☐ Male ☐ Female
2. Age:__________years old
3. Which year are you currently in at the college?
   ☐ First (1st) year ☐ Second (2nd) year ☐ Third (3rd) year ☐ Forth (4th) year

Part II: Knowledge

1. Which infectious disease have you ever heard of? (multiple answers allowed)

☐ Strongyloidiasis ☐ Angiostrongyliasis ☐ Toxoplasmosis ☐ Taeniasis (tape worm)
☐ Leptospirosis ☐ Japanese encephalitis ☐ Dengue fever. ☐ Malaria
☐ Tick-borne diseases ☐ *Opisthorchis viverrine* (liver fluke)
☐ Soil transmitted helminthes/ hook worm/ round worm
☐ None of the above

2. Which of the following are zoonotic infectious diseases? (multiple answers allowed)

☐ Strongyloidiasis ☐ Angiostrongyliasis ☐ Toxoplasmosis ☐ Taeniasis (tape worm)
☐ Leptospirosis ☐ Japanese encephalitis ☐ Dengue fever ☐ Malaria
☐ Tick-borne diseases ☐ *Opisthorchis viverrin* (liver fluke)
☐ Soil transmitted helminthes/ hook worm/ round worm
☐ None of the above

3. How do you get infected with diseases No.1 to No.11?
Choose the appropriate route(s) of infection from the list (a. to f.) and check the corresponding cell, as shown in the example. <u>(Multiple answers allowed)</u>

Route / Diseases	**a.** Eating undercooked/raw fish	**b.** Eating undercooked/raw meat	**c.** Eating unwashed vegetables	**d.** Touching duty soil/water	**e.** Walking barefoot outside	**f.** Bitten by vectors (mosquitoes/ ticks)
**(example) Tetanus**				✓		
**1.** Strongyloidiasis	1					
**2.** Angiostrongyliasis						
**3.** Toxoplasmosis						
**4.** Taeniasis (tape worm)						
**5.** Leptospirosis						
**6.** Japanese encephalitis						
**7.** Dengue fever						
**8.** Malaria						
**9.** Tick borne diseases						
**10.** *Opisthorchis viverrine* (liver fluke)						
**11.** Soil transmitted helminthes/ hook worm/ round worm						

4. Which symptoms may appear if you get infected with infectious diseases No. 1 to No. 11? Choose the symptoms from the list (a. to h.) and check the corresponding cells, as shown in the example. (Multiple answers allowed)

Symptoms / Diseases	**a.** Fever	**b.** Respiratory symptoms (cough, stuffy nose, sore throat)	**c.** Digestive disorders (abdominal pain, diarrhea, vomiting, nausea)	**d.** Headache	**e.** Anemia	**f.** Skin symptoms (purpura, itchiness, rash, scar)	**g.** Muscle pain	**h.** Urinary symptoms (proteinuria, oliguria, kidney function failure)
**(example) Tetanus**							✓	
**1.** Strongyloidiasis								
**2.** Angiostrongyliasis								
**3 .**Toxoplasmosis								
**4.** Taeniasis (tape worm)								
**5.** Leptospirosis								
**6.** Japanese encephalitis								
**7.** Dengue fever								
**8.** Malaria								
**9.** Tick borne diseases								
**10.** *Opisthorchis viverrine* (liver fluke)								
**11.** Soil transmitted helminthes/ hook worm/ round worm								

5. From who did you learn basic hygiene techniques, such as washing hands and gargle? (Multiple answers allowed)

☐ Never learned.

☐ Family members ☐ Friends/Acquaintances

☐ Kindergarten/nursery teachers ☐ Primary school teachers

☐ Secondary school teachers ☐ Others (please specify) : ___________

6. How do you obtain information about infectious diseases (transmission routes, symptoms, and prevention, etc.)? (multiple answers allowed)

☐ Newspaper ☐ Magazine/ journal ☐ Books ☐ TV programs
☐ Radio ☐Internet ☐ Family members ☐ Friends/acquaintances
☐ Primary/secondary school teachers/ classes ☐ University/ collage teachers/ lectures
☐ Hospital/health center
☐ Others (please specify) : ___________

7. From whom do you think it is best to learn basic hygiene techniques?

☐ Family members
☐ Friends/Acquaintances
☐ Kindergarten/Nursery teachers
☐ Primary/Secondary school teachers
☐ Others (please specify): _________________________

Part III: Attitudes

1. I believe there is a possibility of being infected by parasites in my daily life.
   ☐ Disagree ☐ Neutral ☐ Agree
2. I am afraid of getting infectious diseases.
   ☐ Disagree ☐ Neutral ☐ Agree
3. I try to prevent getting infectious diseases in my daily life.
   ☐ Disagree ☐ Neutral ☐ Agree
4. I try to prevent getting or spreading infectious diseases when I attend clinical training at a hospital or clinic.
   ☐ Disagree ☐ Neutral ☐ Agree
5. I think it is important to have knowledge about infectious diseases.
   ☐ Disagree ☐ Neutral ☐ Agree
6. I think it is necessary to provide health education on infectious diseases.
   ☐ Disagree ☐ Neutral ☐ Agree
7. I would like to conduct health education for infectious disease prevention when I become a nurse.
   ☐ Disagree ☐Neutral ☐ Agree
8. I believe people's attitudes toward infectious diseases will change after receiving health education.
   ☐ Disagree ☐ Neutral ☐ Agree
9. I think I may get an infection by eating unwashed vegetables.
   ☐ Disagree ☐ Neutral ☐ Agree
10. I think I may get an infection by eating undercooked or raw fish.
   ☐ Disagree ☐ Neutral ☐ Agree
11. I think I may get an infection by eating undercooked or raw meat.
   ☐ Disagree ☐ Neutral ☐ Agree

12. I think I may get an infection if I don't wash my hands properly.

☐ Disagree ☐ Neutral ☐ Agree

13. I think I may get an infection by walking outside barefoot.

☐ Disagree ☐ Neutral ☐ Agree

14. I think I may get an infection by exposing my skin while walking in forests or grassy areas.

☐ Disagree ☐ Neutral ☐ Agree

15. I think I may get an infection if I don't prevent mosquito bites.

☐ Disagree ☐ Neutral ☐ Agree

16. I think I may get an infection from close contact with pets (ex. bathing together, sleeping together).

☐ Disagree ☐ Neutral ☐ Agree

17. I think I may get an infection by leaving pet feces in the yard or vegetable garden.

☐ Disagree ☐ Neutral ☐ Agree

18. Which infectious diseases do you think should receive more attention in your residential area? (Multiple answers allowed)

☐ Strongyloidiasis
☐ Angiostrongyliasis
☐ Toxoplasmosis
☐ Taeniasis (tapeworm)
☐ Leptospirosis
☐ Japanese encephalitis
☐ Dengue fever
☐ Malaria
☐ Tick-borne diseases
☐ *Opisthorchis viverrini* (liver fluke)
☐ Soil-transmitted helminths/ Hookworm / Roundworm
☐ HIV/AIDS
☐ Sexually transmitted diseases (STD)
☐ Influenza
☐ Food poisoning (bacterial/viral)

☐ None of the above

19. Which infectious diseases do you think will become important in the future? (Multiple answers allowed)

☐ Strongyloidiasis
☐ Angiostrongyliasis
☐ Toxoplasmosis

☐ Taeniasis (tapeworm)
☐ Leptospirosis
☐ Japanese encephalitis
☐ Dengue fever
☐ Malaria
☐ Tick-borne diseases
☐ Opisthorchis viverrini (liver fluke)
☐ Soil-transmitted helminths/ Hookworm / Roundworm
☐ HIV/AIDS
☐ Sexually transmitted diseases (STD)
☐ Influenza
☐ Food poisoning (bacterial/viral)

☐ None of the above

Part IV: Practice

1. Do you try to prevent getting or spreading infectious diseases when you go for clinical training at a hospital or clinic?
   ☐ No
   ☐ Yes
   ➞ *If yes, which of the following measures do you practice?* Multiple answers allowed)
   ☐ Wearing a mask
   ☐ Vaccination
   ☐ Washing hands/ gargling before and after training
   ☐ Others (please specify): ____________________________

2. How often do you eat fresh vegetables?
   ☐ I do not eat fresh vegetables. *(➞ Skip to Question 5)*
   ☐ Every day
   ☐ 2 to 3 times per week
   ☐ 2 to 3 times per month
   ☐ 2 to 3 times per year
3. How do you wash vegetables when you eat them uncooked?
   ☐ I do not wash them
   ☐ Wash with tap water

☐ Wash with pooled water

☐ Others (please specify): ________________________

4. What kinds of vegetables do you eat most often? (Multiple answers allowed)

☐ Lettuce

☐ Holy basil

☐ Hoary basil

☐ Spring onion

☐ Coriander

☐ Chinese cabbage

☐ Spiritweed / Long coriander

☐ Kitchen mint

☐ Heart leaf

☐ Others (please specify): ________________________

5. How often do you eat undercooked or raw meats?

☐ I do not eat undercooked/raw meats. *(➞ Skip to Question 7)*

☐ Every day

☐ 2 to 3 times per week

☐ 2 to 3 times per month

☐ 2 to 3 times per year

6. What kinds of meat do you generally eat, and how do you cook each type?
Please select one option for each type of meat:

Beef:	☐ I do not eat	☐ Raw	☐ A part of the meat is raw	☐ Well cooked
Pork:	☐ I do not eat	☐ Raw	☐ A part of the meat is raw	☐ Well cooked
Chicken:	☐ I do not eat	☐ Raw	☐ A part of the meat is raw	☐ Well cooked
Goat:	☐ I do not eat	☐ Raw	☐ A part of the meat is raw	☐ Well cooked

Others (please specify): ________________________

☐ Raw ☐ A part of the meat is raw ☐ Well cooked

7. How often do you eat undercooked or raw fish?

☐ I do not eat undercooked/raw fish. *(➞ Skip to Question 9)*

☐ Every day

☐ 2 to 3 times per week

☐ 2 to 3 times per month

☐ 2 to 3 times per year

8. Which types of fish do you eat undercooked or raw? (Multiple answers allowed)

   ☐ *Pa-khao mon (Puntius brevis)*

   ☐ *Pa-xiew (Esomus metallicus)*

   ☐ *Tapian Kao (Barbonymus altus)*

   ☐ *Saiton Takao (Cyclocheilichthys armatus)*

   ☐ *Kui Lam Sa (Labiobarbus siamensis)*

   ☐ *Kra soop jud (Hampala dispar)*

   ☐ Others (please specify): ________________________

9. Do you wash your hands before eating?

   ☐ No ☐ Yes, with water only ☐ Yes, with soap

10. Do you wash your hands after using the toilet?

   ☐ No ☐ Yes, with water only ☐ Yes, with soap

11. Do you walk outside barefoot?

   ☐ No ☐ Yes

12. Do you try to avoid skin exposure (ex. wearing long sleeves) when walking in forests or grassy areas?

   ☐ No ☐ Yes

13. Do you use insect repellent?

   ☐ No, I do not use it

   ☐ Yes

   ➞ *If yes, in which situations do you use repellent?* (Multiple answers allowed)

   ☐ Entering mountainous areas ☐ Camping

   ☐ Farming ☐ Garden farming/weeding

   ☐ Others (please specify): ________________________

14. Which insects are you targeting when using repellent? (Multiple answers allowed)

   ☐ Ticks ☐ Mosquitoes

   ☐ Others (please specify): ________________________

15. Have you ever been bitten by ticks?

   ☐ No ☐ Yes ☐ I don't know

16. How often are you bitten by mosquitoes during the mosquito-prevalent season?

   ☐ Very rarely bitten ☐ Once or twice per month

   ☐ Once or twice a week ☐ Almost every day

17. Do you have any pets in your home/living area?

☐ I do not have any pets *(➞ Skip to Question 22)*

☐ Yes

➞ *Which pets do you have?* (Multiple answers allowed)

☐ Dog (number: ___)

☐ Cat (number: ___)

☐ Others (specify the animal and number): ___________________________

18. Do you wash your hands after touching pets?

☐ No ☐ Yes, with water only ☐ Yes, with soap

19. Do you wash your hands after cleaning up pet feces?

☐ No ☐ Yes, with water only ☐ Yes, with soap

20. Do you have any livestock in your home/living area?

☐ I do not have any livestock *(➞ Questionnaire ends. Thank you very much!)*

☐ Yes

➞ *Which livestock do you have?* (Multiple answers allowed)

☐ Cow (number: ___)

☐ Pig (number: ___)

☐ Chicken (number: ___)

☐ Goat (number: ___)

☐ Buffalo (number: ___)

☐ Others (specify the animal and number): ___________________________

21. Do you know how livestock feces are treated at your home?

☐ I do not know

☐ Just leave as they defecate

☐ Treat by burning or purification

☐ Use as fertilizer

☐ Others (please specify): ___________________________

Thank you very much for your cooperation.
